# Non-Genetic Modifiers of Synaptic Plasticity and Neurotransmission in the Central Nervous System (CNS) in Health and Disease

**DOI:** 10.3390/ijms232012135

**Published:** 2022-10-12

**Authors:** Andreas M. Grabrucker

**Affiliations:** 1 Cellular Neurobiology and Neuro-Nanotechnology Lab, Department of Biological Sciences, University of Limerick, V94PH61 Limerick, Ireland; andreas.grabrucker@ul.ie; 2 Bernal Institute, University of Limerick, V94PH61 Limerick, Ireland; 3 Health Research Institute (HRI), University of Limerick, V94PH61 Limerick, Ireland

This Special Edition intends to focus on the influence of non-genetic factors as modifiers of synaptic plasticity and neurotransmission in health and disease. Among the factors discussed in this Issue are traumatic brain injuries, overactivation due to seizures, diet, particularly zinc status and high-fat diet (HFD), and the presence of drugs such as ketamine. These non-genetic factors and their interplay with genetic factors influence how our brain develops and functions, regulating behavior and mental well-being. However, despite an increasing understanding of how genes and their encoded proteins contribute to synapse formation, plasticity, and function in the central nervous system (CNS), the interplay between non-genetic factors and these synaptic components remains poorly understood.

Seizures can have genetic and non-genetic causes, as well as environmental triggers. Febrile seizures (FSs) in early life that can be triggered by fever affect brain development and function, so that the risk of developing neurological disorders and cognitive impairment in later life is significantly increased. To better understand the underlying mechanisms, Postnikova et al. [1] analyzed morphological and functional changes in the hippocampus of their FS model (young rats exposed to hyperthermia). Cell numbers decreased in the CA1 and hilus but not in the CA3 or dentate gyrus areas of the hippocampus. Along with these morphological alterations, long-term potentiation (LTP) in CA3-CA1 synapses was strongly reduced [1]. This was attributed to the lower *N*-methyl-D-aspartate receptor (NMDAR) signaling caused by insufficient activation of the glycine site of NMDARs. Thus, these results reveal a new molecular mechanism how FSs impact brain development. Seizure prevention is, therefore, critical to healthy brain development. Pawlik et al. [2] could show that the seizures observed in young rats (pilocarpine-induced) were delayed and attenuated by pretreatment with a non-convulsive dose of methionine sulfoximine (MSO). Their study reveals that MSO affects seizure activity by interfering with synaptic glutamate (Glu)-release but not with Glu recycling [2].

Dietary factors are other key non-genetic factors that interact with genetic factors. In this Special Issue, Oberto et al. report that the exposure of male Neuropeptide Y Receptor Y1 (Npy1r)*^rfb^* mice (with conditional inactivation of the *Npy1r* gene in forebrain principal neurons) to an HFD increased body weight growth, adipose tissue, blood glucose levels and caloric intake for these mice compared to male controls [3], while female mice were only marginally affected. Thus, forebrain NPY-Y1Rs may mediate the susceptibility to obesity in male and female mice with low levels of gonadal hormones [3], illustrating how an HFD may lead to alternative outcomes based on the CNS genetic background.

Dietary zinc availability, in particular, seems critical to healthy brain development and function. Zinc was reported to be an essential factor in the development and treatment of major depressive disorder (MDD). For example, monoamine-based antidepressants mobilize zinc in the blood and brain of depressed patients and animal models. Pochwat et al. investigated the effects of NMDAR antagonists (ketamine and Ro 25-6981) in the absence of zinc [4]. NMDARs binds zinc, which acts as an inhibitor. Both Ro 25-6981 and ketamine normalized depressive-like behaviors in zinc-deficient rats, revealing that both NMDAR antagonists act independently of the zinc regulation of NMDAR subunits.

While acute zinc deficiency in adolescents and adults may be linked to MDD, early-life zinc deficiency has been associated with an increased risk of developing autism spectrum disorders (ASD). This is nicely illustrated in a review article by Sauer et al. in this issue [5]. The review summarizes findings from animal studies using prenatal zinc-deficient mice that display neurobiological and ASD-like behavioral abnormalities and highlights potential underlying pathomechanisms. Notably, the data show that zinc signaling is mechanistically linked to ASD-associated genetic factors such as synaptic SH3 and multiple ankyrin repeat domains 3 (SHANK3). In addition, the review provides evidence that prenatal zinc deficiency may underlie several environmental risk factors associated with ASD. Thus, the levels of zinc need to be tightly controlled at synapses. In line with this, De Benedictis et al. report the presence of specific family members and their subcellular localization of zinc transporters (ZnTs) and Irt-like proteins (ZIPs) in the rat brain [6]. Interestingly, the authors revealed ZIP4 localization at synapses and found the zinc transporter in a complex with SHANK3.

SHANK3 is a major scaffolding protein of the postsynaptic density, whose dynamics can be regulated by zinc. The protein mediates plastic changes in excitatory synapses within the CNS. This becomes evident after brains are subjected to another non-genetic factor: mild traumatic brain injury (mTBI). Urrutia-Ruiz et al. report that mTBI triggered a loss of hippocampal excitatory synapses with a partial time-dependent recovery in wild-type mice. However, in the absence of SHANK3 (using *Shank3*∆*11−/−* mice), no significant synaptic alterations were detected due to the lack of structural synaptic plasticity in *Shank3* knockout mice [7]. However, these mice had fewer excitatory synapses at baseline. Given that mutations in SHANK3 are linked to ASD, this loss of synaptic plasticity may explain some of the ASD-like behavioral impairments of these mice, such as problems adjusting to new situations and cognitive deficits [7].

In conclusion, non-genetic factors such as stressors, nutrition, physical activity, or drugs can affect the CNS, particularly synapse plasticity and neurotransmission, through a variety of mechanisms, ranging from modulation of the microbiome (gut–brain-signaling), triggering or preventing chronic/acute (neuro)inflammation, to directly altering the function of synaptic proteins (Figure 1). In many cases, several of the aforementioned processes will occur in parallel. Therefore, all the environmental factors impacting at the synaptic level have not yet been deciphered. However, understanding how non-genetic factors contribute to neuronal dysfunction may lead to novel treatment and prevention strategies.

## Figures and Tables

**Figure 1:**
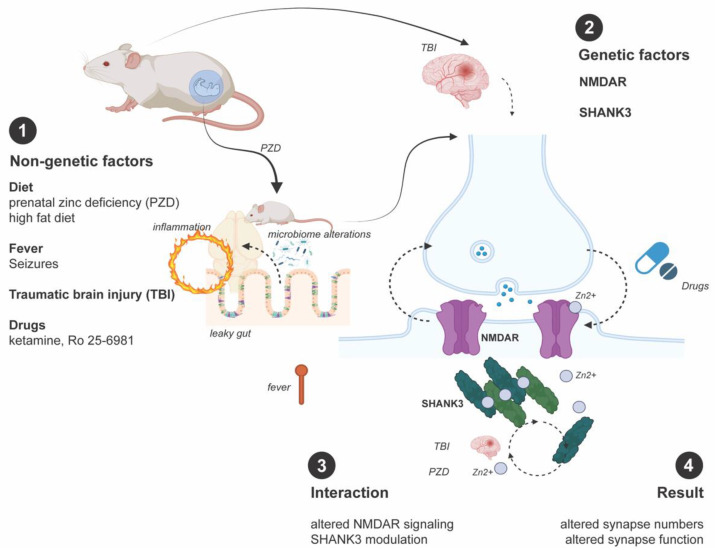
Some of the many possible interactions between non-genetic factors and the synapses discussed in articles in this Special Issue, affecting synapse formation and plasticity. Altered synaptic functions will ultimately cause, contribute to, or modify the physiology or pathology of the CNS.
